# Metabolic Reprogramming in the Heart and Lung in a Murine Model of Pulmonary Arterial Hypertension

**DOI:** 10.3389/fcvm.2018.00110

**Published:** 2018-08-15

**Authors:** Jose L. Izquierdo-Garcia, Teresa Arias, Yeny Rojas, Victoria Garcia-Ruiz, Arnoldo Santos, Silvia Martin-Puig, Jesus Ruiz-Cabello

**Affiliations:** ^1^CIC biomaGUNE, San Sebastian-Donostia, Spain; ^2^CIBER de Enfermedades Respiratorias, Madrid, Spain; ^3^Centro Nacional de Investigaciones Cardiovasculares, Madrid, Spain; ^4^Unidad de Gestion Clinica del Corazon, Hospital Universitario Virgen de la Victoria, Málaga, Spain; ^5^IKERBASQUE, Basque Foundation for Science, Bilbao, Spain; ^6^Universidad Complutense Madrid, Facultad de Farmacia, Departamento de Quimica en Ciencias Farmaceuticas, Madrid, Spain

**Keywords:** pulmonary arterial hypertension, metabolomics, preclinical models, molecular imaging, NMR spectroscopy

## Abstract

A significant glycolytic shift in the cells of the pulmonary vasculature and right ventricle during pulmonary arterial hypertension (PAH) has been recently described. Due to the late complications and devastating course of any variant of this disease, there is a great need for animal models that reproduce potential metabolic reprograming of PAH. Our objective is to study, *in situ*, the metabolic reprogramming in the lung and the right ventricle of a mouse model of PAH by metabolomic profiling and molecular imaging. PAH was induced by chronic hypoxia exposure plus treatment with SU5416, a vascular endothelial growth factor receptor inhibitor. Lung and right ventricle samples were analyzed by magnetic resonance spectroscopy. *In vivo* energy metabolism was studied by positron emission tomography. Our results show that metabolomic profiling of lung samples clearly identifies significant alterations in glycolytic pathways. We also confirmed an upregulation of glutamine metabolism and alterations in lipid metabolism. Furthermore, we identified alterations in glycine and choline metabolism in lung tissues. Metabolic reprograming was also confirmed in right ventricle samples. Lactate and alanine, endpoints of glycolytic oxidation, were found to have increased concentrations in mice with PAH. Glutamine and taurine concentrations were correlated to specific ventricle hypertrophy features. We demonstrated that most of the metabolic features that characterize human PAH were detected in a hypoxia plus SU5416 mouse model and it may become a valuable tool to test new targeting treatments of this severe disease.

## Introduction

Pulmonary arterial hypertension (PAH) is a rare form of pulmonary hypertension that primarily affects the lung microvasculature. In PAH, the gradual obliteration of the arterial lumen results in a progressive increase in pulmonary vascular resistance, leading to right ventricle (RV) dysfunction and death (1). The pulmonary vascular pathology of PAH is characterized by a major remodeling of small arteries and arterioles due to the proliferation of resident and migrating inflammatory cells. Mitochondrial dysfunction in these cells plays an essential role in the pathogenesis of the disease (2, 3). Pulmonary artery smooth muscle and endothelial cells of PAH patients proliferate and exhibit a shift in glucose metabolism from oxidative phosphorylation to glycolysis (4, 5). Mitochondrial dysfunction has been reported in human PAH lung samples (6) and human pulmonary endothelial cells genetically mutated to reflect PAH-related traits (7). In parallel with the increase in pulmonary vascular resistance, RV myocytes develop a mitochondrial metabolic switch similar to that observed in lung samples (8). Metabolic adaptations in the heart for improving RV function involve cellular and molecular events that ultimately affect glycolysis and fatty acid oxidation (9). In accordance with these results, some authors have suggested a common metabolic remodeling in the vascular cells of the pulmonary circulation and extrapulmonary tissues (10, 11). Therefore, the study of the metabolic reprogramming in PAH as a multiorgan event may reveal several key metabolic targets that are directly involved in PAH pathogenesis and may form the basis of biomarker and drug discovery programs.

One of the primary difficulties for PAH biomarker development and drug discovery programs is the absence of any preclinical model that recapitulates most of the clinical features of human PAH (12, 13). Hence, experimental animal models that display a clustering of proliferated cells in the lumen of pulmonary arteries are essential to monitor the molecular mechanisms of the disease. Among the described PAH models, the mouse model that combines hypoxia exposure with the inhibition of vascular endothelial growth factor receptor (VEGF) using Semaxanib (SU5416) has been used to study several of the etiologic mechanisms involved in cell hyperproliferation (14, 15). This PAH model develops a more profound and sustained pulmonary hypertension phenotype than that induced by 3 weeks of chronic hypoxia alone (14) and is characterized by neointimal smooth muscle cell proliferation and obliteration, leading to increased RV pressure, RV hypertrophy, and evidence of incipient heart failure (15). Due to the relevance of this process in the cardiopulmonary progression of PAH, the aim of this study was to characterize the metabolic changes associated with cell proliferation in this mouse model of PAH. To achieve this objective, energy metabolism was studied *in vivo* by (^18^F)-2-fluoro-2-deoxy-D-glucose (FDG) positron emission tomography (PET). Intact lung and heart tissues were also analyzed by high resolution magic angle spinning (HR-MAS) nuclear magnetic resonance (NMR) spectroscopy.

## Materials and methods

### Animals and ethical approval

The Madrid Government Animal Care and Use Committee approved all experimental protocols (Proex 381/15). All animals were treated in accordance with the guidelines approved by the Spanish scientific procedures act (32/2007) and European Union Directive 86/609/EEC. The study was performed using an established model of PAH in mice that is generated by hypoxia exposure combined with Semaxanib (SU5416) administration (HPX+SU model). Healthy normoxic mice (NMX group) and healthy hypoxic mice (HPX group) were used as controls. The HPX+SU murine PAH model has been well-characterized in previous studies (14–16). Briefly, ten-weak-old male C57BL/6 mice (Charles River Laboratories) were exposed to normobaric hypoxia (10% of oxygen) for 3 weeks (*n* = 25) and were only removed from the chamber once per week for the administration of subcutaneous injections of the VEGF inhibitor, SU5416 (MedChem Express. Stockholm, Sweden). SU5416 was suspended in carboxymethyl cellulose (CMC) (0.5% [w/v] CMC sodium, 0.9% [w/v] sodium chloride, 0.4% [v/v] polysorbate 80, 0.9% [v/v] benzyl alcohol in deionized water) and injected at 20 mg/kg. HPX mice (*n* = 12) were exposed to the same hypoxic conditions and weekly sham injections. NMX mice (*n* = 25) were maintained in a room with normal oxygen levels.

Mice were monitored weekly to check for changes in body weight, external physical appearance or the presence of labored respiration. After 3 weeks, mice were euthanized using a ketamine-xylazine overdose (300 and 30 mg/kg, respectively). Lung tissue was exsanguinated and immediately collected for histological and/or metabolomic profiling. The heart was removed, and RV hypertrophy (Fulton's index) was measured by weighing the RV relative to the left ventricle (LV) plus septum (LV+S). RV and LV samples were snap frozen for metabolomic analysis.

### Histology

Lung tissue samples were preserved for 24 h after collection in 10% formalin, stored in 70% ethanol, and then paraffin-embedded for histological studies. Paraffin-embedded lung tissue sections of 4-μm thickness were stained with Verhoeff-van Gieson to measure the medial thickness of the lung arteries or with picrosirius red to measure lung collagen deposition. Medial wall thickness was measured using the equation described by Vitali et al. (14). Lung macrophage infiltration was determined by immunohistochemistry using the F4/80 antibody. All sections were digitally imaged and analyzed by a NanoZoomer Digital Pathology Imaging system (NDP, Hamamatsu. Japan). Picrosirius red-stained sections were also analyzed using polarized light in a Nikon ECLIPSE 90i upright microscope (with NIS-Elements 3.22.11 acquisition software). Presented values are the mean of 10 fields taken from 10 lung sections per mouse (*n* = 8 per group).

### Echocardiography

Echocardiographic studies were conducted before (basal condition) and 3 weeks after exposure to normoxia/hypoxia conditions. Mice were anesthetized using a 1–2% isoflurane/O_2_ gas mixture. Cardiac function (ejection fraction), chamber dilatation, and wall thickness were analyzed by transthoracic echocardiography using a Vevo 2100 system and a 45-MHz probe (Visualsonics, Toronto, Canada). Images were taken by a blinded, experienced operator, and measurements were performed offline by two experienced readers. The mice were placed on a heating pad under light anesthesia, and sevoflurane levels (~1%) were adjusted to obtain a target heart rate of ~500 bpm. 2D and M-mode parasternal echocardiographic long-axis and short-axis views at three levels were recorded to obtain accurate measurements.

Color and pulse-wave Doppler images were acquired by positioning images for the LV at the tip of the pulmonary valve leaflets and aligned to show maximum laminar flow. Angled parasternal long-axis views were used to study pulmonary artery flows. Integrals of pulmonary flow, pulmonary acceleration times and RV ejection times were measured to estimate RV systolic pressure (17). These values were averaged across a minimum of five cardiac cycles (*n* = 8 per group).

### Positron emission tomography and computed tomography (PET/CT) imaging

*In vivo* FDG uptake was analyzed using a dual-head PET combined with CT (Bioscan system, manufactured by Mediso). Mice were fasted overnight prior to scanning, and blood glucose levels were tested prior to contrast agent injection. Nuclear imaging was conducted prior to the induction of hypoxia (basal condition) and after one, 2 and 3 weeks of hypoxia (*n* = 8 per group). The animals were anesthetized using a 1–2% isoflurane/O_2_ gas mixture administered through a nose mask and maintained at a temperature of approximately 30°C from the time of the injection until the scans were completed.

FDG contrast agent was obtained from the PET Technological Institute (ITP) (Madrid, Spain). After measuring the exact concentration in a radioisotope calibrator, FDG (21.07 ± 1.094 MBq) was intravenously administered 1 h before PET imaging.

OsiriX Software was used to analyze the PET/CT sequences. For FDG uptake in the RV, LV and lungs, three-dimensional regions of interest (3D-ROIs) were drawn for each tissue, and the maximum standardized uptake value (SUV) was quantified.

### NMR data acquisition

Intact lung, RV and LV tissue samples were examined by HR-MAS NMR using a Bruker AMX500 spectrometer (11.7 T). Samples were placed into a 50-μl zirconium oxide rotor using a rinsed cylindrical insert with 15 μl of a 0.1 mM solution of TSP in deuterium water (D_2_O) and spun at 4,000 Hz to remove the effects of spinning side bands from the acquired spectra. Shimming and NMR preparation times were reduced to a minimum, while the sample was chilled to 4°C to minimize metabolic changes. A number of two-dimensional homonuclear and heteronuclear experiments, such as standard gradient-enhanced correlation spectroscopy (COSY), ^1^H–^1^H total correlated spectroscopy (TOCSY) and gradient-selected heteronuclear single quantum correlation (HSQC), were performed to carry out component assignments. A control ^1^H NMR spectrum was measured between consecutive two-dimensional (2D) spectra. No gross degradation was noted in the signals of multiple spectra acquired under the same conditions.

Standard solvent-suppressed spectra were grouped into 32,000 data points, which were averaged over 256 acquisitions. The data acquisition lasted a total of 15 min using a sequence based on the first increment of the nuclear Overhauser effect spectroscopy (NOESY) pulse sequence to suppress the effects of water. Sample acquisition was performed using a spectral width of 8,333.33 Hz prior to Fourier transformation, and the free induction decay (FID) signals were multiplied by an exponential weight function corresponding to a line broadening of 0.3 Hz. The spectra were referenced to the TSP singlet at a chemical shift of 0 ppm.

The NMR spectra were processed as described previously (18). Briefly, ^1^H-NMR spectra were data-reduced to equal-length integral segments (δ = 0.01 ppm), and they were normalized to the total sum of the spectral regions.

### Statistical analysis

The required sample size for imaging studies was calculated using G Power software (19). The variables were compared using two-tail Student's *t*-tests and are expressed as the mean ± standard deviation (SD).

### Metabolomics profiling

Principal components analysis (PCA) (20) was applied to full NMR spectra in order to extract the most discriminative spectral subset from the total pool of metabolites and to remove outliers. The data obtained from this analysis were centered and scaled. The potential statistically significant differences NMR areas selected from the PCA loading matrix were confirmed by Hoteling's T2 test (21). A minimum of 10 samples per group were included in the PCA analysis to guarantee a classification error lower than 10%. Multivariate statistical analysis was performed with the Metabonomic package (rel.3.3.1) (22). In addition, the resonances highlighted as significantly different by the PCA loading matrix were identified and individually integrated for metabolic quantification using the Global Spectral Deconvolution algorithm of MestRenova v. 8.1 (Mestrelab Research S.L., Santiago de Compostela, Spain). The resonances were identified according to the Human Metabolome Database (23), and characteristic cross-peaks from 2D spectra to help in unequivocal assignation of these metabolites. For metabolic quantification, statistical significance was determined using a Bonferroni-corrected Student's *t*-test (24) assuming unequal variance, and *p* < 0.05 was considered statistically significant.

## Results

### Pulmonary hypertension characterization

**Figure 1:**
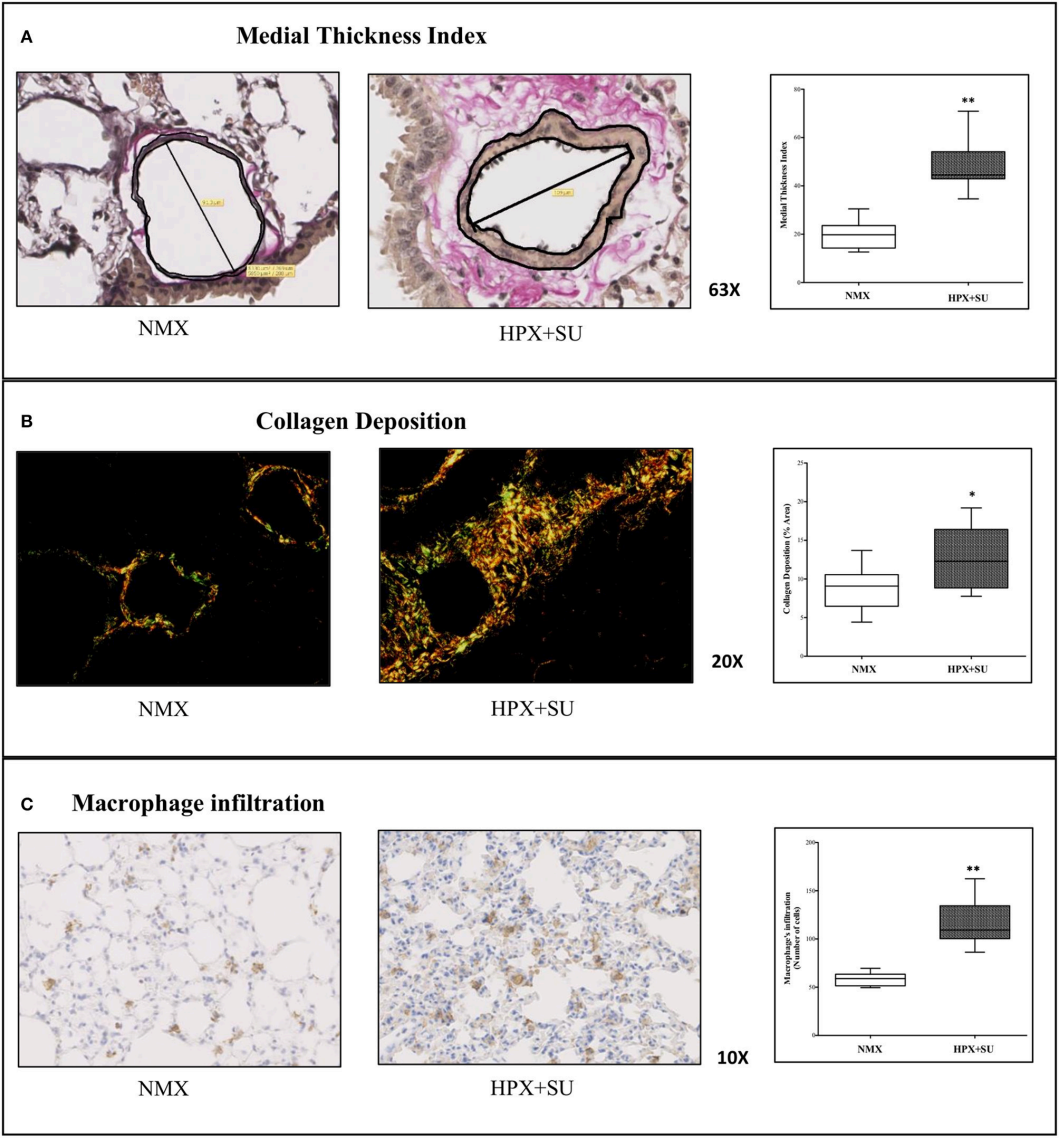
Hypoxia and SU5416 treatment induces pulmonary vascular remodeling. **(A)** Representative pulmonary arterioles stained with Verhoeff-van Gieson stain (upper) and medial wall thickness index values (lower). **(B)** Representative picture of picrosirius red staining (upper) and percentage of lung collagen deposition (lower). **(C)** Representative picture of macrophage infiltration using the F4/80 antibody (upper) and macrophage quantification (lower). Statistical differences (^*^P < 0.05, ^**^P < 0.01) were detected using an analysis of variance and two-tailed Student's *t*-test.

Vascular remodeling was confirmed in HPX+SU mice, which displayed a significant increment in medial wall blood vessel thickness (Figure 1A) compared to NMX mice (*P* < 0.0001). Compared to NMX mice, a substantial deposition of collagen was detected in the vascular media and the lung parenchyma from HPX+SU (*P* < 0.05) (Figure 1B). HPX+SU mice also exhibited macrophage infiltration (*P* < 0.001) (Figure 1C).

**Figure 2:**
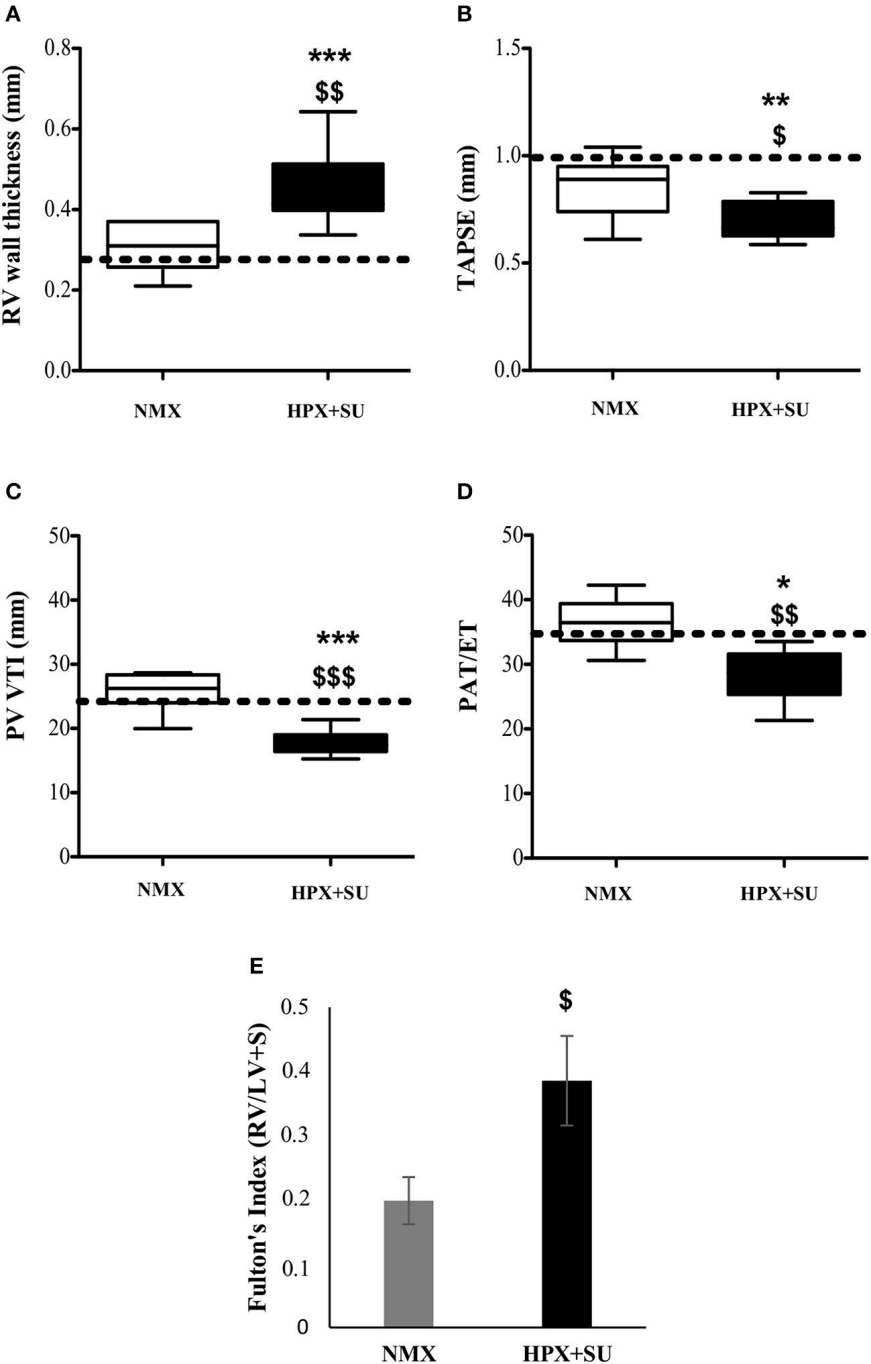
Right Ventricle (RV) weight and echocardiographic measurements change after hypoxia and SU5416 treatment. **(A)** RV wall thickness. **(B)** Tricuspid annular plane systolic excursion (TAPSE). **(C)** The velocity–time integral across the pulmonary valve (PV-VTI). **(D)** Ratio of pulmonary acceleration time (PAT) to ejection time (ET). **(E)** Fulton's index. The dotted line indicates basal conditions. Statistical differences were detected using an analysis of variance and two-tailed Student's *t-*test (^*^P < 0.05; ^**^P < 0.01; ^***^P < 0.001 vs. basal conditions. ^$^*P* < 0.05; ^$$^*P* < 0.01; ^$$$^*P* < 0.001 vs. NMX group). HPX+SU = hypoxia plus SU5416 group, *n* = 8; NMX = normoxia group, *n* = 12.

RV wall thickness (Figure 2A) was increased in HPX+SU mice after 3 weeks of hypoxia (*P* < 0.001 vs. basal conditions, *P* < 0.01 vs. NMX). The tricuspid annular plane systolic excursion (TAPSE) was significantly decreased in the HPX+SU group (*P* < 0.01 vs. basal conditions, *P* < 0.05 vs. NMX) (Figure 2B). The velocity–time integral across the pulmonary valve (PV-VTI) was also significantly decreased in HPX+SU mice (*P* < 0.01 vs. basal conditions, *P* < 0.001 vs. NMX) (Figure 2C). The ratio of pulmonary acceleration time (PAT) relative to ejection time (ET) was significantly reduced in HPX+SU versus NMX mice and basal conditions (*P* < 0.05 vs. basal conditions, *P* < 0.01 vs. NMX mice) (Figure 2D). Finally, RV weight was significantly increased in HPX+SU mice compared with NMX mice after 3 weeks of hypoxia (*P* < 0.05) (Figure 2E).

### Pulmonary metabolic reprogramming

**Figure 3:**
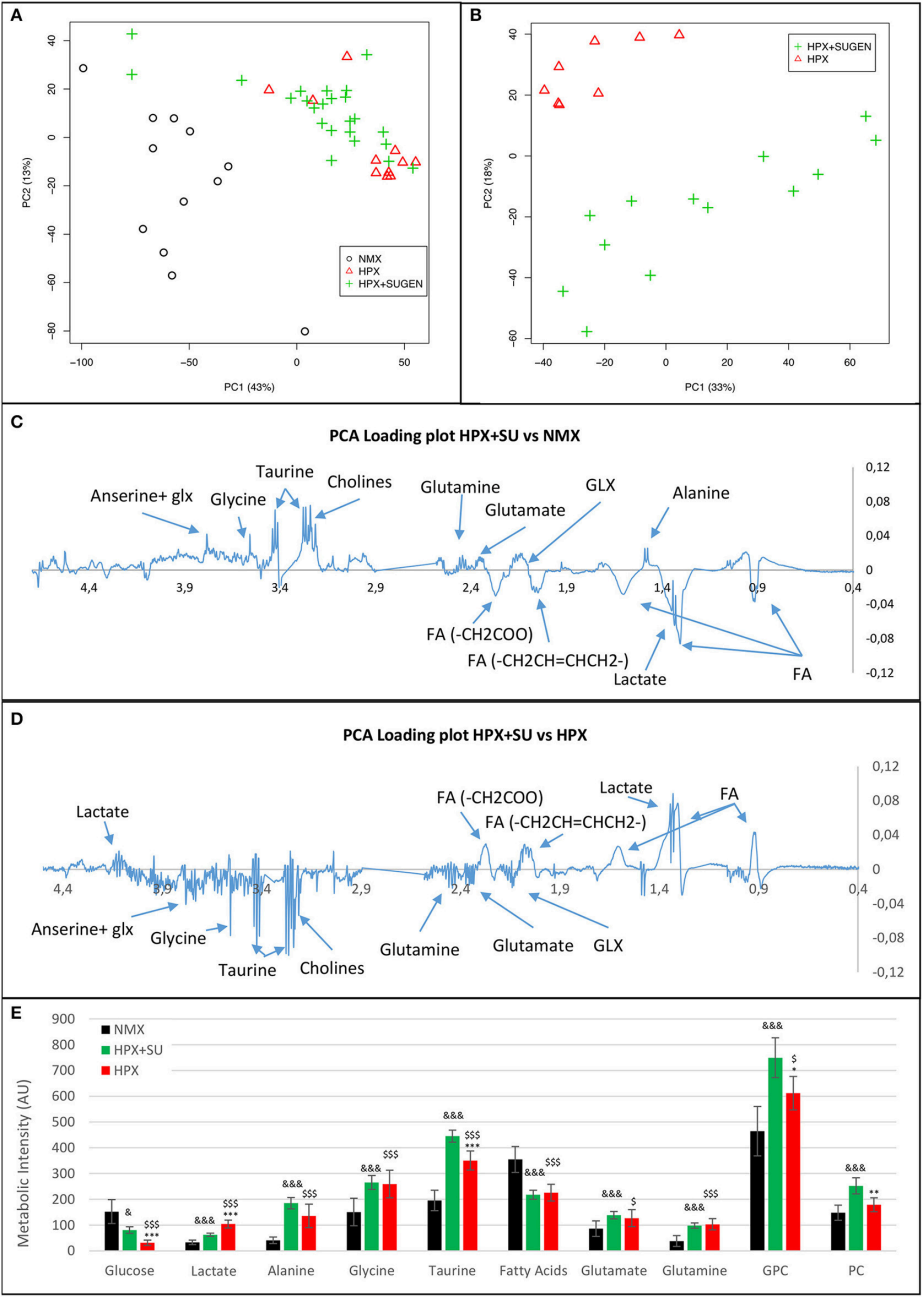
Metabolomic profiling of lung samples. **(A)** Principal components analysis (PCA) performed on ^1^H-MRS data from lung reveals a clear separation between NMX, HPX, and HPX+SU groups. **(B)** Second PCA reveals also a clear separation between HPX and HPX+SU groups. PCA loading plots identify metabolites that discriminate between NMX and HPX+SU groups **(C)** and between HPX and HPX+SU groups **(D)**. The resonance that best distinguished the groups was selected based on a significance level lower than 5.00e-02 by Hoteling's T2 test. **(E)** Metabolites with the potential to distinguish the groups were quantified. NMX (*n* = 15); HPX+SU (*n* = 22); HPX (*n* = 12). Statistical significance was determined using a Bonferroni-corrected Student's *t*-test (HPX+SU vs. HPX: ^*^P < 0.05; ^**^P < 0.01; ^***^P < 0.001. HPX+SU vs. NMX: ^&^*P* < 0.05; ^&&&^*P* < 0.001. HPX vs. NMX: ^$^*P* < 0.05; ^$$$^*P* < 0.001). PC, principal component; AU, arbitrary units; GPC, glycerophosphocholine; GLX, glutathione; FA, fatty acid.

**Figure 4:**
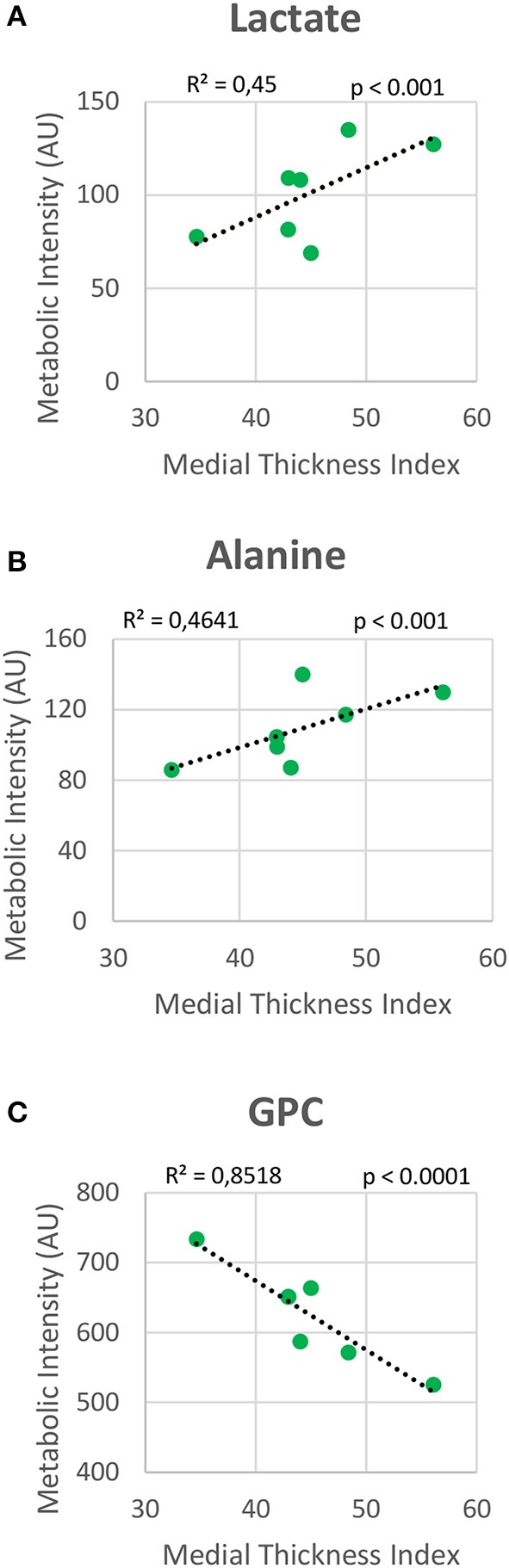
Correlation plot between lung metabolic intensities of lactate **(A)**, alanine **(B)**, and glycerophosphocholine (GPC) **(C)**, and medial thickness index. Correlations were assessed using Pearson's R-squared correlation coefficient (*R*^2^).

Metabolomic profiling of lung samples discriminated between NMX and both HPX and HPX+SU groups (Figure 3A). A slight difference between HPX+SU and HPX mice was also detected and further confirmed after removing NMX group from the PCA analysis, showing a clear separation between HPX+SU and HPX mice (Figure 3B). The PCA loading plots highlight the most significant metabolites that distinguish between groups (Figures 3C,D). Specifically, we observed a reduction in the concentration of glucose and free fatty acids, and an increase in lactate, alanine, glycine, glutamate, glutamine, taurine, glycerophosphocholine (GPC), and phosphocholine concentrations in HPX and HPX+SU mice compared to NMX mice (Figure 3E, Table 1). When we compared HPX+SU mice to HPX mice, we found significant differences in glucose and lactate concentrations. Lactate, alanine, and glycerophosphocholine concentrations showed a good correlation with the histologic features of PAH such as arterial medial thickness index (Figure 4).

**Table 1:** Summary of postulated metabolites and their relative amounts from NMX, HPX, and HPX+SU groups.

	**NMR region (PPM)**	**Mean (SD)**			**T Test**		
		**NMX**	**HPX+SU**	**HPX**	**HPX+SU vs. NMX**	**HPX+SU vs. HPX**	**HPX vs. NMX**
**LUNG TISSUE**							
Alanine	1.48	40.76 (12.75)	184.89 (22.02)	135.65 (45.92)	2,13E-16	8,89E-02	3,40E-11
FFA	1.60	354.60 (50.46)	217.79 (17.45)	225.73 (32.72)	7,70E-04	7,41E-01	2,81E-04
Glutamate	2.32	86.00 (30.01)	138.58 (14.02)	126.92 (33.01)	3,22E-05	1,45E-01	1,42E-02
Glutamine	2.42	37.95 (21.82)	97.93 (10.34)	102.84 (21.82)	2,32E-08	3,63E-01	1,68E-08
PC	3.23	147.99 (29.58)	252.24 (31.57)	178.69 (26.98)	3,39E-04	4,80E-03	1,49E-01
GPC	3.24	464.68 (95.98)	749.34 (77.53)	611.94 (65.25)	7,07E-04	2,52E-02	2,05E-02
Taurine	3.28	195.38 (39.57)	445.08 (23.65)	350.44 (37.05)	6,14E-09	1,63E-03	6,37E-06
Glycine	3.54	150.64 (53.72)	265.66 (26.92)	259.14 (53.46)	2,31E-06	7,26E-01	6,27E-06
Lactate	4.09	33.31 (8.76)	62.25 (6.15)	104.07 (15.52)	1,54E-04	5,22E-04	2,24E-08
Glucose	4.63	152.18 (46.59)	80.30 (12.65)	31.48 (10.32)	2,68E-02	2,25E-05	3,67E-05
**RIGHT VENTRICLE TISSUE**							
Alanine	1.48	90.13 (17.28)	101.35 (29.09)	122.55 (14.82)	1,07E-01	5,01E-02	1,36E-03
FFA	1.6	899.89 (65.87)	754.23 (77.44)	848.48 (51.32)	5,31E-06	3,68E-03	7,40E-02
Glutamate	2.32	121.49 (19.00)	147.15 (31.66)	121.08 (11.04)	8,27E-04	2,25E-02	9,67E-01
Glutamine	2.42	62.51 (19.35)	127.41 (40.83)	149.99 (19.68)	3,03E-08	1,26E-01	1,05E-07
Creatine Phosphate	3.02	335.63 (70.54)	465.43 (112.72)	438.53 (64.95)	1,12E-04	5,13E-01	7,62E-03
Taurine	3.28	688.61 (115.61)	1111.28 (212.79)	947.41 (131.30)	1,53E-08	4,69E-02	4,51E-04
Glycine	3.54	13.10 (5.09)	30.28 (5.89)	18.34 (9.46)	5,12E-06	1,71E-03	2,04E-02
Lactate	4.09	101.41 (19.79)	202.85 (32.33)	186.76 (36.36)	4,98E-09	2,85E-01	8,45E-09
Glucose	4.63	45.42 (12.07)	12.94 (13.96)	13.08 (7.10)	9,39E-08	9,78E-01	9,27E-07
**LEFT VENTRICLE TISSUE**							
Alanine	1.48	121.70 (24,57)	137.76 (50,27)	104.56 (23.23)	3,76E-01	6,31E-02	1,17E-01
FFA	1.6	272.83 (47.88)	210.38 (112.55)	186.13 (85.88)	1,24E-01	5,83E-01	1,10E-01
Glutamate	2.32	99.96 (33.73)	114.42 (36.00)	126.93 (42.21)	3,66E-01	4,76E-01	1,25E-01
Glutamine	2.42	178.32 (42.56)	190.60 (56.92)	215.87 (45.01)	5,92E-01	2,71E-01	6,50E-02
Creatine Phosphate	3.02	400.50 (93.26)	437.37 (154.63)	447.15 (148.78)	5,27E-01	8,84E-01	4,06E-01
Taurine	3.28	1028.10 (173.68)	911.39 (327.87)	987.20 (274.78)	3,33E-01	5,71E-01	6,92E-01
Lactate	4.09	131.25 (26.33)	213.50 (43.91)	186.23 (32.86)	7,81E-05	1,21E-01	4,84E-04

**Figure 5:**
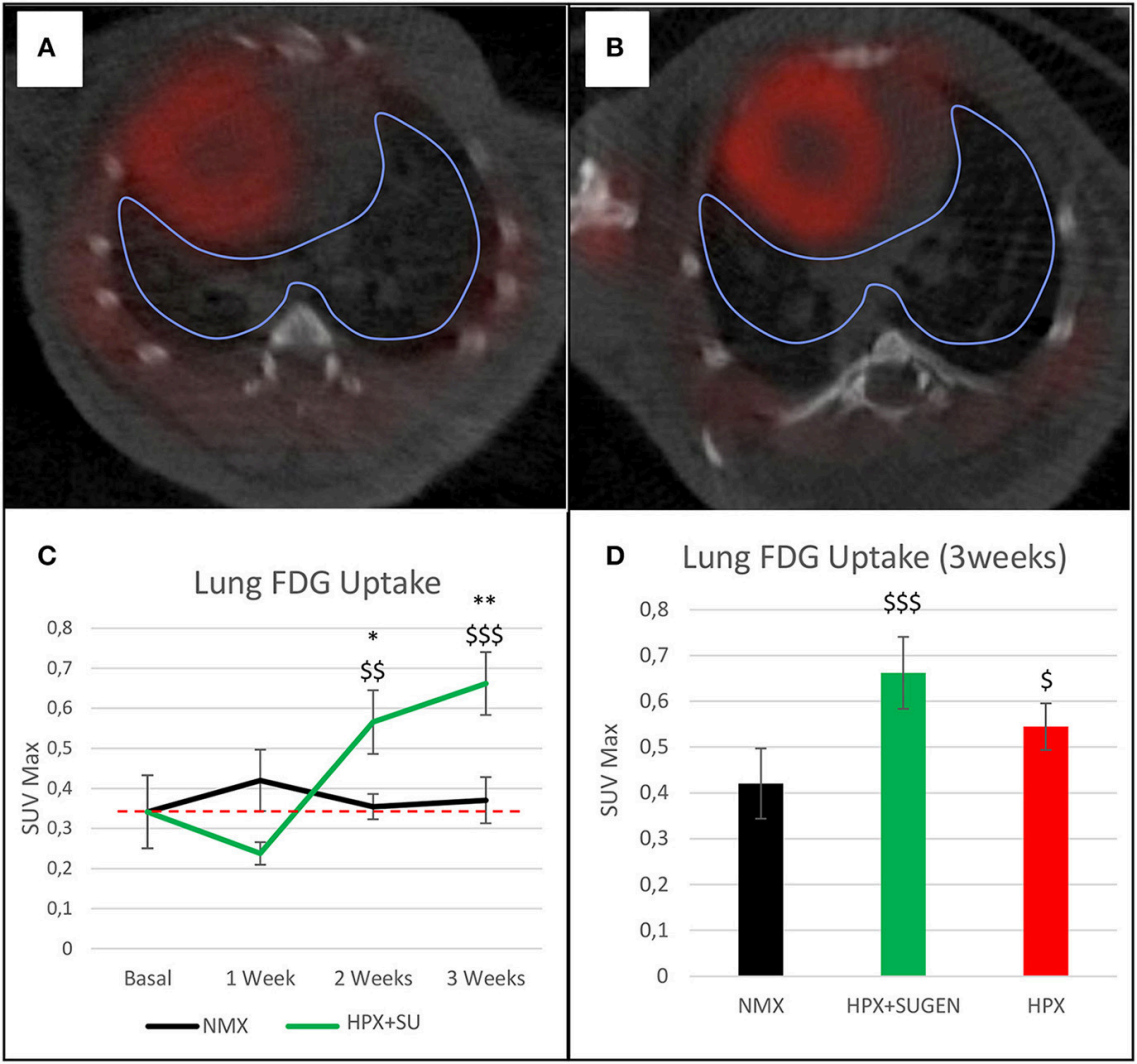
Fused positron emission tomography (PET)/computed tomography (CT) images of the lung parenchyma. Representative images of NMX **(A)** and HPX+SU **(B)** mice at the end of the 3-week protocol. ^18^F-FDG uptake in HPX+SU mice significantly increased after the first week vs. the initial prehypoxic exposure conditions and compared to NMX mice **(C)**. Standardized uptake value maximum (SUVmax) at 3-weeks also shows an increase in ^18^F-FDG uptake in HPX+SU and HPX groups **(D)**. The dotted line indicates basal conditions. Statistical differences were determined by analysis of variance with a two-tailed Student's *t*-test (^*^P < 0.05; ^**^P < 0.01 vs. basal conditions. ^$^*P* < 0.05;^$$^*P* < 0.01; ^$$$^*P* < 0.001 vs. NMX group).

The altered or adapted energy metabolism was confirmed by *in vivo* PET imaging (Figures 5A,B). ^18^F-FDG uptake in HPX+SU mice significantly increased after the first week vs. the initial pre-hypoxic exposure conditions and compared to values for NMX mice (Figure 5C). At the end of the protocol for up to 3 weeks of exposure, HPX+SU and HPX mice showed significantly higher ^18^F-FDG uptake than NMX mice (Figure 5D). HPX+SU also showed higher (no significant) ^18^F-FDG uptake than HPX mice.

### Cardiac metabolic reprogramming in PAH models

**Figure 6:**
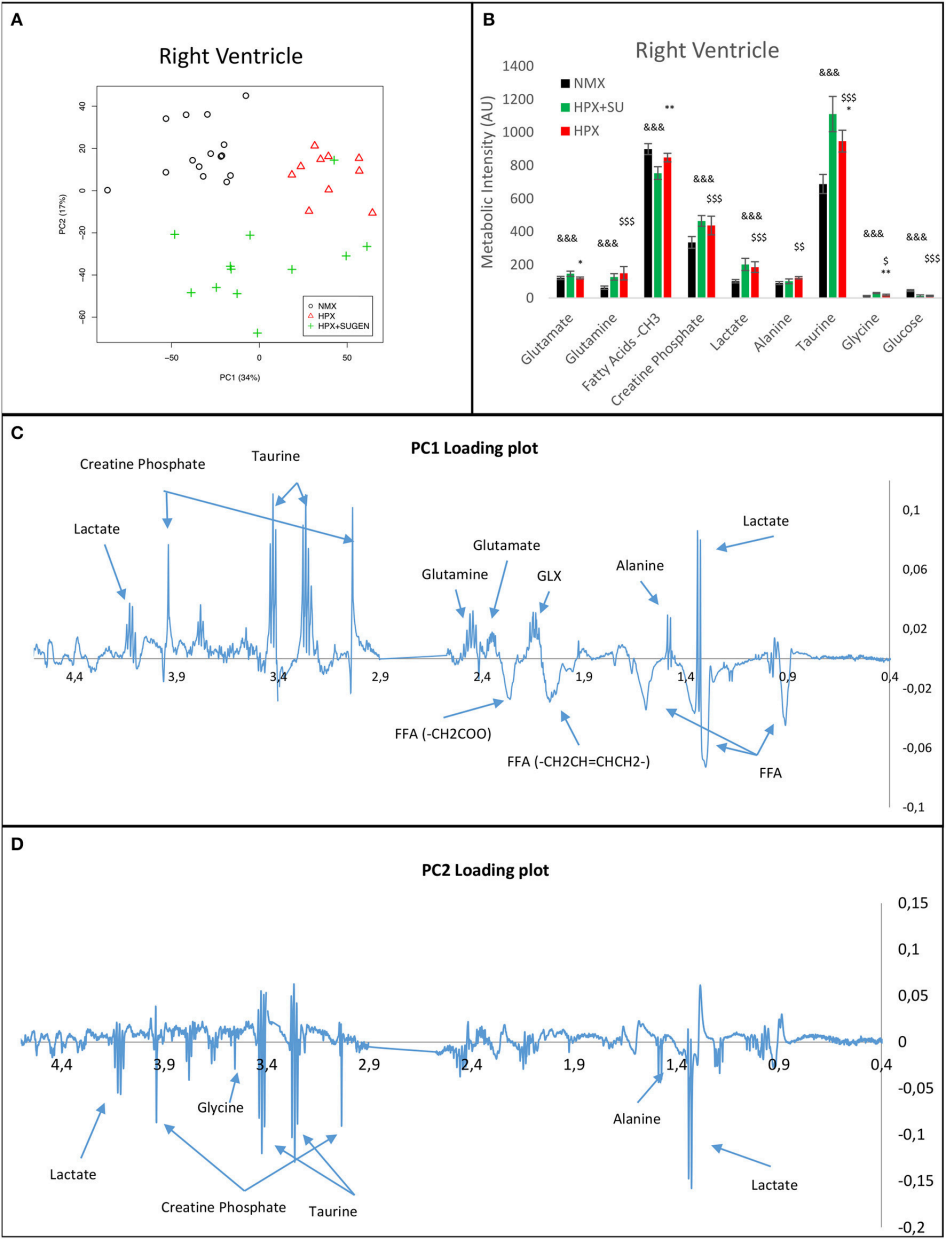
Metabolomic profiling of right ventricle samples. Principal components analysis (PCA) performed on ^1^H-MRS data from lung reveals a clear separation between NMX, HPX, and HPX+SU groups **(A)**. PC1 **(C)** and PC2 **(D)** loading plots identify metabolites that discriminate between the groups. The resonance that best distinguished the groups was selected based on a significance level lower than 5.00e-02 by Hoteling's T2 test. Metabolites with the potential to distinguish the groups were quantified **(B)**. NMX (*n* = 15); HPX (*n* = 10); HPX+SU (*n* = 11). Statistical significance was determined using a Bonferroni-corrected Student's *t*-test (HPX+SU vs. HPX: ^*^P < 0.05; ^**^P < 0.01. HPX+SU vs. NMX: ^&&&^*P* < 0.001. HPX vs. NMX: ^$^*P* < 0.05; ^$$^*P* < 0.01; ^$$$^*P* < 0.001). PC, principal component; AU, arbitrary units; GLX, glutathione; FA, fatty acid.

**Figure 7:**
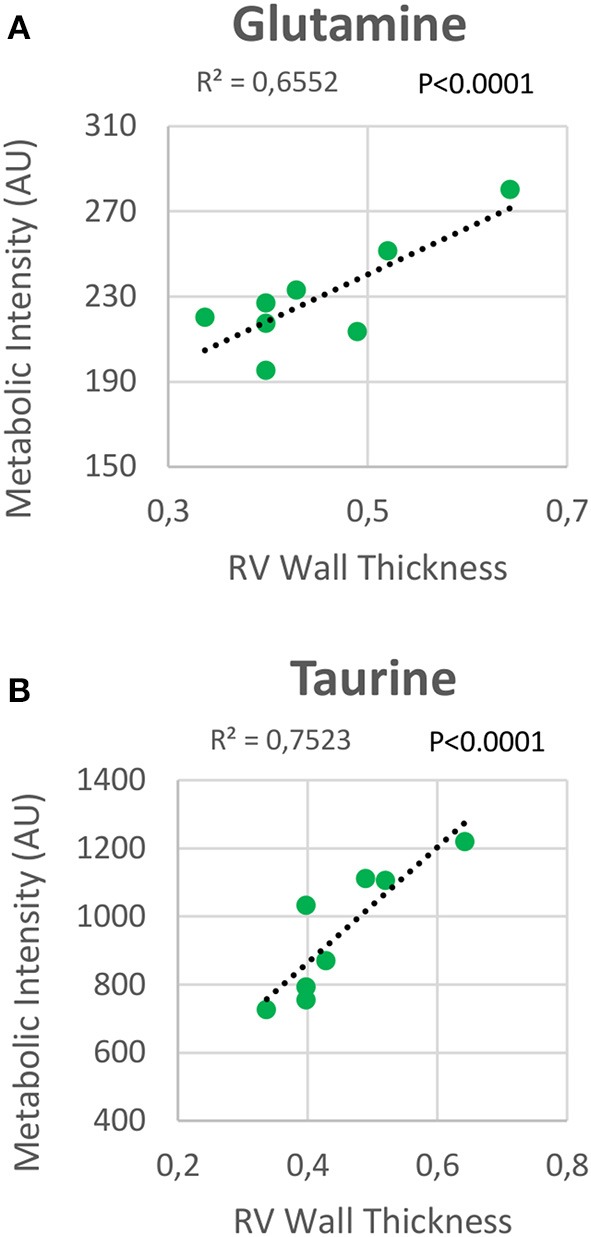
Correlation plot between the right ventricle (RV) metabolic intensities of glutamine **(A)** and taurine **(B)**, and the RV thickness. Correlations were assessed using Pearson's R-squared correlation coefficient (*R*^2^).

RV NMR spectra provided a nearly perfect discrimination between the three groups along the first two principal components (Figure 6A). The PCA loading plots show the metabolites that changed between groups (Figures 6C,D). We found a shift in energy metabolism, with higher metabolic concentrations of glutamine, creatine phosphate, lactate, taurine and glycine, and lower concentration of glucose in HPX+SU and HPX groups (Figure 6B, Table 1). Focusing on the differences between HPX+SU and HPX groups, we found that taurine, glycine, and glutamate concentrations were significantly lower in HPX group, whereas free fatty acids and alanine concentrations were increased. We also confirmed the correlation between metabolic data and echocardiography parameters. Glutamine and taurine intensities showed a strong correlation with RV wall thickness (Figure 7).

**Figure 8:**
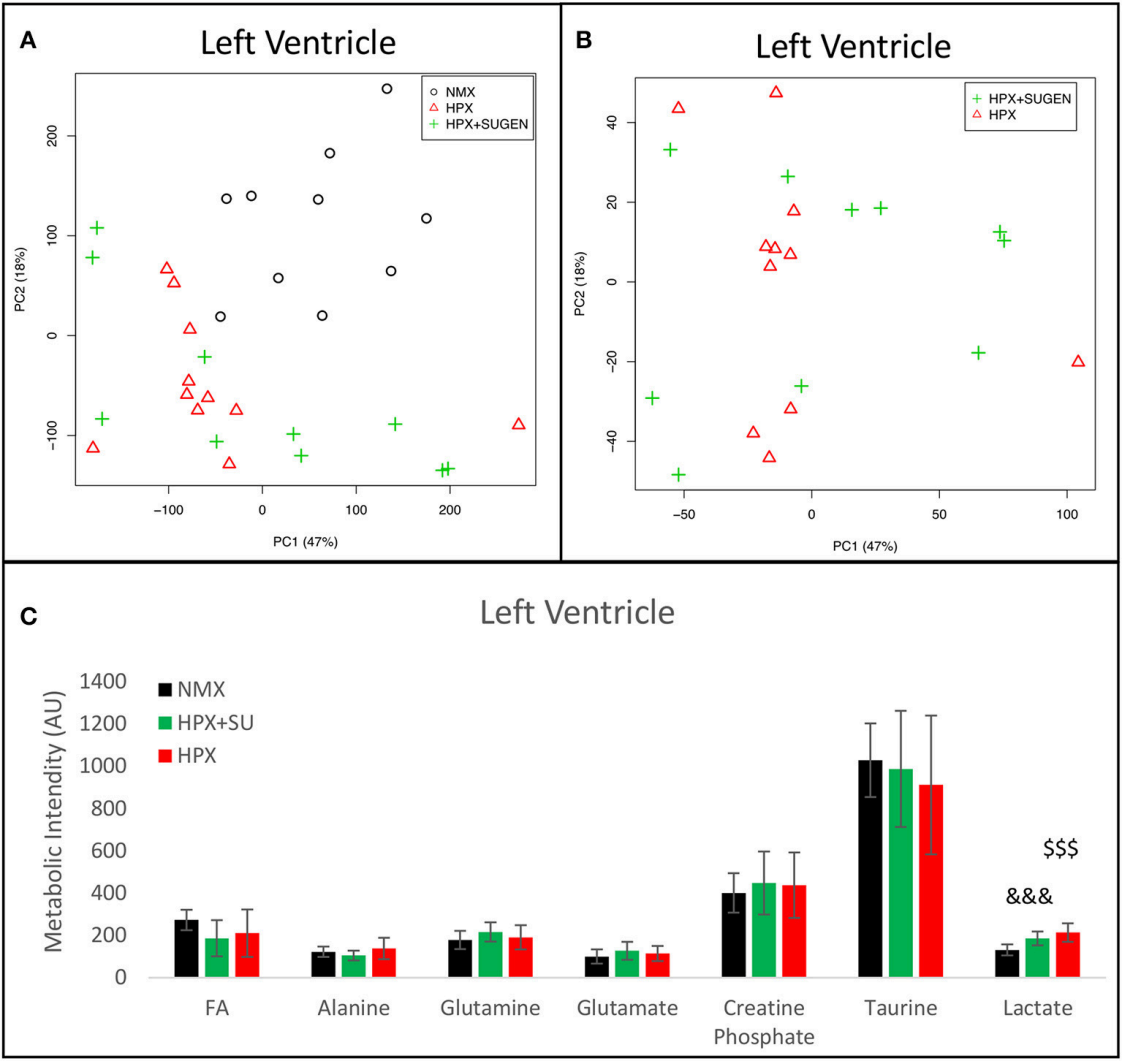
Metabolomic profiling of left ventricle (LV) samples. **(A)** Principal components analysis (PCA) performed on ^1^H-MRS data from LV reveals a clear separation between NMX, HPX, and NMX+SU groups. **(B)** Second PCA cannot show a separation between the hypoxia (HPX) and hypoxia plus SU5416 treatment (HPX+SU) groups. **(C)** Metabolic quantification in left ventricle tissue. NMX (*n* = 10); HPX+SU (*n* = 10); HPX (*n* = 11). Statistical significance was determined using a Bonferroni-corrected Student's *t*-test (HPX+SU vs. NMX: ^&&&^*P* < 0.001. HPX vs. NMX: ^$$$^*P* < 0.001). AU, Arbitrary Units.

Metabolomic analysis of LV samples indicated a much less acute metabolic effect in this ventricle than in the RV. PCA scores plot (Figure 8A) shows the discrimination between NMX and both hypoxic groups, but not between HPX+SU and HPX mice (Figure 8B). We detected higher lactate signals in the HPX and HPX+SU groups, but concentrations of other metabolites are not significantly different (Figure 8C, Table 1).

**Figure 9:**
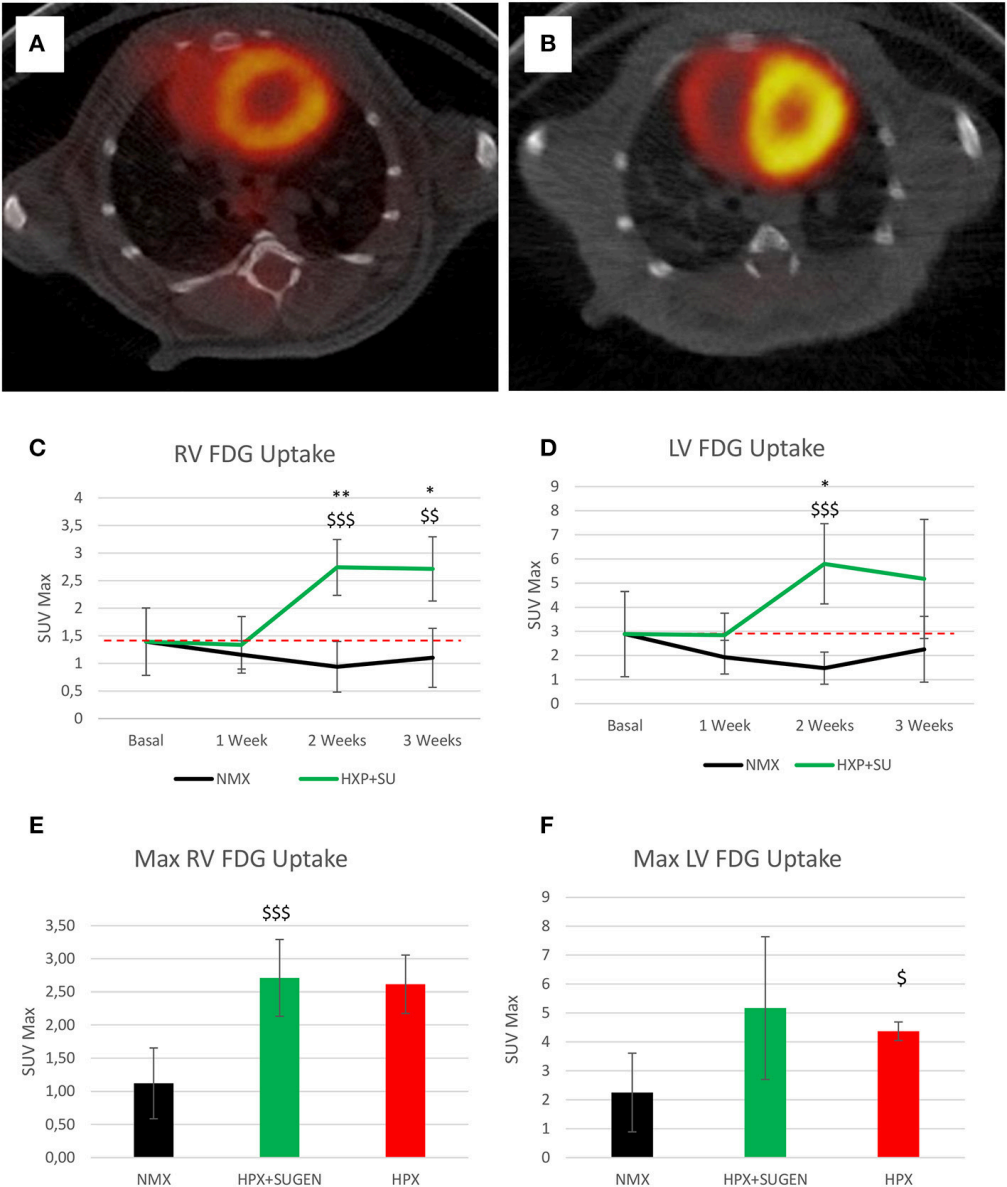
Fused positron emission tomography (PET)/computed tomography (CT) images of the left and right ventricles. Representative images of NMX **(A)** and HPX+SU **(B)** mice at the end of the 3-week protocol. ^18^F-FDG uptake in HPX+SU mice significantly increased after the first week vs. the initial prehypoxic exposure conditions and compared to NMX mice in the right **(C)** and left **(D)** ventricles. Standardized uptake value maximum (SUVmax) at 3-weeks also shows an increase in ^18^F-FDG uptake in HPX+SU and hypoxia (HPX) groups in the right ventricle **(E)** and HPX group in the left ventricle **(F)**. The dotted line indicates basal conditions. Statistical differences were determined by analysis of variance with a two-tailed Student's *t*-test (^*^P < 0.05; ^**^P < 0.01 vs. basal conditions. ^$^*P* < 0.05; ^$$^*P* < 0.01; ^$$$^*P* < 0.001 vs. NMX group).

Finally, ^18^F-FDG uptake was significantly increased in HPX+SU ventricles compared to uptake under basal conditions and in NMX mice (Figures 9A,B) after the first week of hypoxia exposure (Figures 9C,D). After 3 weeks under hypoxia, HPX+SU and HPX mice showed higher ^18^F-FDG uptake than NMX mice in both ventricles (Figures 9E,F).

## Discussion

Robust animal models that reproduce the pathological features of human PAH are needed to improve our understanding of the pathobiology of PAH, particularly in a dynamic scenario, in order to develop new therapeutic strategies that target alternative pathways in PAH. Here, we characterized the metabolic profile of an established PAH mouse model using NMR-based metabolomics and ^18^F-FDG PET imaging. PET imaging has been used previously to characterize PAH in animal models (25, 26) and patients (27, 28), and the ^1^H-MRS analysis of intact tissues provides an excellent tool for understanding biochemical processes associated with diseases (29). It should be noted that previous metabolomic studies on PAH were performed in cellular models (7), in plasma samples of PAH patients (30–32), in human lung tissue obtained at the time of lung transplantation (6) and in RV tissue from a PAH rat model (33). To the best of our knowledge, this is the first metabolomic study designed to assess the metabolic reprogramming associated with PAH in lung and heart tissues from a mouse model.

We chose a murine model of PAH that combines hypoxia exposure and VEGF inhibition because it addresses most of the etiologic mechanisms involved pulmonary vascular cell hyperproliferation. Hence, this model could serve to identify subtle metabolic changes specifically associated with smooth muscle cell proliferation and subsequent RV hypertrophy. We confirmed the development of human PAH features in our mouse model. HPX+SU mice showed a significant increase in blood vessel medial wall thickness, deposition of collagen in the vascular media and macrophage infiltration. We also confirmed RV hypertrophy (Fulton's index) and dysfunction by echocardiography. Thus, our results are consistent with PAH clinical data (34) and with data from previous studies in animal models (14, 15). However, previous studies using a mouse model of hypoxia and VEGF inhibition (14, 15) did not probe the metabolic profiles of the animals, which was the focus and a major strength of our study.

**Figure 10:**
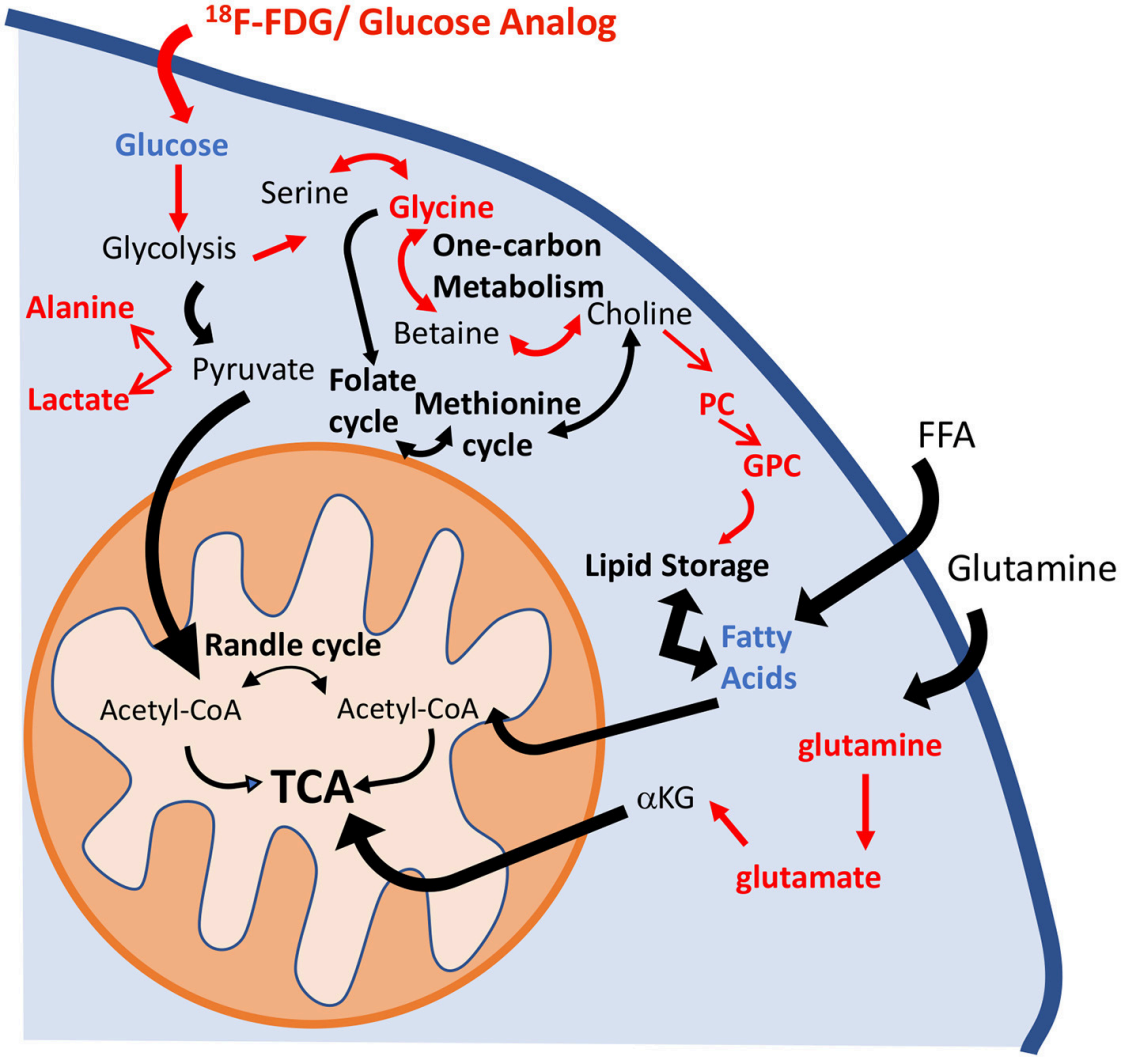
Schematic illustration of the metabolic pathways found to be altered in the lung parenchyma. Enhancement of the glycolytic pathway, including an increase in pulmonary FDG uptake, higher lactate and alanine concentrations, and a decrease in the pulmonary glucose concentrations in HPX+SU mice. The increased glycine concentration is characteristic of an upregulation of one-carbon metabolism. Higher levels of phosphocholine and glycerophosphocholine also point to an increase in lipid biosynthesis. Glutaminolysis was increased to support maybe TCA activity. For clarity, increased pathways/metabolites are displayed in red, decreased pathways/metabolites are displayed in blue, and some intermediate metabolic steps are not illustrated. PC, phosphocholine; GPC, glycerophosphocholine; FFA, free fatty acids; αKG, α-ketoglutarate.

Metabolic reprogramming in PAH is now recognized as a major contributor to the pathogenesis of pulmonary vascular disease (35). The pulmonary vasculature in PAH displays a normoxic activation of hypoxia-inducible factor 1-alpha (HIF-1α), which creates a “pseudo-hypoxic” environment despite normal oxygen availability (36). One of the consequences of HIF-1α activation is a metabolic shift toward aerobic glycolysis. The “Warburg effect,” or the upregulation of glycolysis in the presence of oxygen, has been described in the development of the PAH (4). Previous studies have shown that HIF-1α activates over 100 genes involved in the development of hypoxic pulmonary hypertension (8, 37). Specifically, Kim et al. (38) reported the upregulation of glucose transporters (GLUT1 and GLUT3) and of pyruvate dehydrogenase kinase 1 and 4 (PDK1 and PDK4), which promote the inhibition of pyruvate dehydrogenase (PDH) activity and block the entrance of pyruvate into the Krebs cycle. These significant alterations induce an increase in glucose uptake and a reduction of glucose flux into the mitochondria. As a consequence, TCA cycle activity is decreased, and the activity of anaplerotic pathways that replenish the intermediates of the TCA cycle is increased (7). Lipid metabolism has also been highlighted as one of the hallmarks of PAH progression. Sutendra et al. (39) showed that the inhibition of fatty acid oxidation due to the absence of malonyl-coenzyme A decarboxylase (MCD) promotes glucose oxidation and prevents the metabolic shift toward glycolysis. Furthermore, metabolic modulators that are used clinically and that mimic the lack of MCD can reverse PAH induced by hypoxia or monocrotaline (39, 40). Our results confirm the presence of these metabolic adaptations in the HPX+SU mouse model (Figure 10). We have identified significant alterations in the glycolytic pathway that are characterized by an increase in pulmonary FDG uptake, higher lactate and alanine concentrations, and a decrease in pulmonary glucose concentrations in HPX+SU mice. We have also confirmed the upregulation of glutamine metabolism, with a significant increase in glutamine and glutamate concentrations in the lungs of HPX+SU mice may maintain mitochondrial functionality. Alterations in lipid metabolism were also confirmed by the reduction of free fatty acid concentration that may indicate an upregulation of beta-oxidation and/or *de novo* lipid biosynthesis.

In addition, we have identified other new significant alterations in glycine and choline metabolism. The reversible interconversion of serine and glycine is directly coupled to one-carbon metabolism and is involved in nucleotide, lipid and protein biosynthesis (41–43). These reactions proceed in a cyclical manner in which a carbon unit is transferred to other metabolic pathways and eventually replenished from several sources. Glycine metabolism integrates metabolic input from central carbon (glycolysis) and nitrogen (glutamine) metabolism, and this pathway has recently been highlighted as essential for the biosynthesis required for cell proliferation (44). In addition, glycine is especially interesting because of its direct anti-inflammatory and anti-angiogenic effects on endothelial cells (45). Aberrant choline metabolism with upregulated levels of GPC has been recognized as a feature of cancer cell proliferation (46). Elevated levels of choline-containing precursors and breakdown products of membrane phospholipids were also detected in pulmonary arterial vascular smooth muscle cells from patients with PAH, pointing to a link between PAH progression and a cancer-like growth pattern (47).

The metabolic alterations detected in RV are in line with the alterations recently published by Graham et al. (48) in a very early mouse model of PAH (SU5416+ 1 week of hypoxia). In the RV, these changes are likely induced by ischemia rather than by impaired oxygen sensing (36). However, the RV undergoes a number of metabolic changes that are transcriptionally mediated. Although the role played by HIF-1α in the metabolic remodeling of the RV in PAH is not completely clear, several investigators have found that HIF-1α is increased in RV hypertrophy models (25, 49). As a result of HIF-1α activation, angiogenesis was increased, and PDH activity was decreased (34). The PDH-mediated metabolic switch to glycolysis is associated with the impairment of RV function, decreased cardiac output and reduced RV contractility (9). Increased lactate production further impairs RV function secondary to acidosis. The shift to the less energetically efficient process of glycolysis results in an increased glucose flux that can be quantified using ^18^F-FDG PET imaging (50). We confirmed these metabolic features in our model of PAH, which showed significantly increased lactate and alanine concentrations in the RV, lower glucose concentration and higher FDG uptake. However, HPX-SU mice also showed increased FDG uptake in the LV and alterations in glycolytic pathway were detected in the HPX control group. Previous studies using hypoxia to induce PAH reported that key enzymes in the glycolytic pathway were upregulated in both ventricles, along with the upregulation of HIF-1α (51, 52), suggesting that changes occurred in response to hypoxia and not to RV overload. We have detected similar features where FDG uptake and lactate production are significantly altered in both HPX+SU and HPX mice. We also detected an alteration in lipid metabolism in the RV characterized by lower concentration of free fatty acids in HPX+SU RV tissue. During hypoxia, free fatty acids are metabolized to *de novo* lipogenesis, with a concomitant reduction in lipid oxidation, resulting in lipid accumulation inside the cells (53, 54).

The increase in glutamine and taurine concentrations in RV tissue are proposed as significant biomarkers of RV hypertrophy in HPX+SU mice. Both metabolites showed a high correlation with RV wall thickness. In a normal heart, the glutaminolysis rate is very low; however, it is selectively induced in the RV in PAH via the c-Myc transcriptional pathway, likely as a consequence of RV ischemia (55). Although further studies are needed to completely understand the function of glutaminolysis in RV hypertrophy, extrapolation from studies of cancer and endothelial cells metabolism in PAH would suggest that glutaminolysis supports rapid myocyte growth in RV hypertrophy. The physiological role of taurine is more difficult to understand (56). Taurine is the most abundant amino acid in the heart and likely plays an important role in heart failure, especially as taurine deficiency results in LV dysfunction (57). Taurine can directly inhibit hypertrophy produced by angiotensin II in ventricular myocytes (58); therefore, taurine accumulation can be considered an adaptation to the development of the disease.

### Limitations

The PAH model combing hypoxia and SU5416 treatment addresses several of the etiologic mechanisms involved in pulmonary vascular cell hyperproliferation (16), although it also presents several limitations. In PAH, normoxic HIF-1α activation in the pulmonary vasculature initiates the metabolic reprogramming. In this animal model, we reproduced this trigger by inducing chronic hypoxia, which activates the HIF-1α pathway not only in the lung but also in the heart and other tissues. Therefore, the metabolic reprogramming observed in the RV will be induced by both pulmonary vasculature overpressure and exposure to chronic hypoxia. The inclusion of a second control group of healthy mice under hypoxia and the analysis of metabolic alterations in the LV allows us to discriminate the metabolic effects of each contribution, but some minor metabolic changes caused by RV overload could be obscured by the metabolic effects of chronic hypoxia. Second, we did not measure RV systolic pressure (RVSP) to avoid the metabolic response to the procedure. Although RVSP is the gold standard for determining pulmonary hypertension in mice, it is determined with a microtip catheter inserted through the jugular vein that may induce a metabolic alteration in the heart. We confirmed the PAH phenotype previously reported in this animal model (14, 15) by measuring Fulton's and medial thickness indices. Finally, further studies are required to completely understand the role of VEGF inhibition in this model. Several lines of evidence indicate that SU5416 may play a critical role in initial endothelial cell death and in the selection of apoptosis-resistant proliferating cells, which eventually obliterate the pulmonary arteries (59).

## Conclusions

We demonstrated that the PAH mouse model induced by the combination of hypoxia and SU5416 treatment reproduces the metabolic abnormalities observed in the RV and pulmonary circulation in PAH patients. Specifically, we detected an upregulation of glycolysis and the presence of glutamine and fatty acid metabolic pathways in lung vasculature. We also detected alterations in some metabolites related to cell proliferation that may represent new therapeutic targets for PAH. In addition, we monitored the specific RV metabolic alterations induced by pulmonary overpressure. In summary, our study demonstrates that metabolomic analysis in a murine model of PAH induced by hypoxia and VEGF inhibition may become a valuable tool for testing new treatments for this severe disease.

## Data availability

The datasets generated for this study can be found in the figshare repository https://figshare.com/s/9823b75c9bf0ff15fc8f.

## Author contributions

JI-G designed studies, performed experiments, analyzed data, and prepared and reviewed the manuscript. TA designed studies, performed experiments, and analyzed data. YR performed experiments. VG-R. analyzed data. AS and SM-P reviewed the manuscript. JR-C designed studies and prepared and reviewed the manuscript.

### Conflict of interest statement

The authors declare that the research was conducted in the absence of any commercial or financial relationships that could be construed as a potential conflict of interest.
